# Isolation and characterization of Hofbauer cells from human term placentas

**DOI:** 10.3389/fimmu.2026.1671127

**Published:** 2026-03-16

**Authors:** Dancan M. Wakoli, Bartholomew N. Ondigo

**Affiliations:** 1 Laboratory of Infectious Diseases, Department of Biochemistry and Molecular Biology, Egerton University, Njoro, Kenya; 2 Centre for Global Health Research, Kenya Medical Research Institute, Kisumu, Kenya

**Keywords:** Hofbauer cells, infectious diseases, isolation, macrophages, maternal autoimmune pathology, placenta

## Abstract

Hofbauer cells (HBCs) are macrophages of fetal origin present in the human placenta that play a key role in maintaining a healthy pregnancy. They are the most abundant immune cells (> 90%) in the placental villi. They have been implicated in various complications of pregnancy, though their role in these pathologies remains rudimentary. In addition, despite being discovered over a century ago, there is no consensus on the specific time window between delivery and placental sample processing for HBCs isolation, the site of biopsy sampling, and isolation protocol. In previous studies, mechanical disintegration, enzymatic digestions, density gradient separation, homogenization, adherence, rosetting, and immune selection techniques have been used to isolate HBCs for immunological studies. Here we review current knowledge on HBCs isolation methods, their role in healthy and pathologic pregnancy, and the impact of pregnancy complications on HBCs population and phenotype. A literature search was conducted on Scopus, PubMed, and Google Scholar databases using specific keywords, including “Isolation of Hofbauer cells, Hofbauer cells and infectious diseases, Hofbauer cells on immune function, and Hofbauer cells and maternal autoimmune pathology.” No restrictions were placed on the year of publication or author affiliation during the selection process. Developing a standardized and harmonized HBCs isolation technique(s) will allow comparative studies across laboratories, research groups studying HBCs. These standardized approaches will provide the necessary impetus for studies on HBCs and their functionality in healthy and placental pathologies.

## 1 Introduction

Hofbauer cells (HBCs) are specialized macrophages present in the human placenta that play a crucial role in maintaining a healthy pregnancy. They are fetal macrophages that form more than 90% of the fetal immune cell population in the placental villi (1–5). Despite the discovery of HBCs over 100 years ago, they remain poorly understood (4, 6). They are located in the stroma within the placental villus (chorionic villus). Hofbauer cells are also found in the amnion and chorion of the placenta (7). They start to appear in the placenta from day 18^th^ after conception and they persist throughout pregnancy (8). However, there is no consensus on the exact time of appearance in pregnancy; earlier studies have shown that they appear from the fourth or sixth week and others have reported their presence from day 10 (9–11). These cells multiply until 18 weeks and diminish as pregnancy progresses to term (12, 13).

Hofbauer cells are thought to originate from multiple sites, and their origin is dependent on the gestation age. In early stages of pregnancy, they originate from the hypoblast-derived yolk sac monocyte progenitor cells and the stroma villous mesenchymal stem cells (14–16). In later stages of pregnancy, they originate from fetal bone marrow monocytes recruited to the placenta (4, 10, 14, 17). Also, it has been argued that during late in pregnancy, the liver monocytes differentiate to give rise to HBCs (18).

The shape of HBCs varies from round, ovoid, to spindle/stellate with their size ranging from 10-30 µM hence they are pleomorphic cells (9, 12). They have small nucleus and very many granulated cytoplasm (12), and short profiles of endoplasmic reticulum (19). They are plastic cells meaning they can change their phenotype in response to different signals and micro environmental stimuli (8, 20). Despite scanty information about their role, they are thought to perform multiple critical roles for successful pregnancy, namely: regulation of placental growth (angiogenesis, tissue remodeling, and vasculogenesis) (8, 15, 21, 22), response to external pathogens that enter the placenta directly and also mount an inflammatory response to maternal diseases such as obesity, preeclampsia, and diabetes (23–26), control of stromal water content, transfer of ions and serum proteins across the maternal-fetal interface (11).

There is no harmonized standard protocol for isolating HBCs. These techniques have several limitations, including reduced purity due to contamination by maternal macrophages (27–29), HBCs activation upon immunoselection (3), loss of surface markers due to long hours of enzymatic digestion that may complicate HBCs characterization, and alteration of the HBCs *in situ* state caused by *in vitro* culturing prior to analysis, which may also promote trophoblast outgrowth and thereby compromising analysis after cell culture (7). HBCs have been implicated in different pregnancy complications such as gestational diabetes, placental malaria, preeclampsia, chorionamnionitis, Zika, HIV, Villitis of Unknown Etiology (VUE), and bacterial infections, though their role remains unknown (8, 30–33). Here we review current knowledge on HBCs isolation, their role in healthy and pathologic pregnancy, and the impact of pregnancy complications on HBCs population, function, and phenotype.

## 2 Diversity of Hofbauer cells and their role in healthy pregnancy

Macrophages produces distinct functional phenotypes as a response to signals and micro environmental stimuli in order to perform their functions, this process is called polarization (34–37). The macrophages can polarize into either classically activated phenotype (M1) or alternatively activated ones (M2). The two phenotypes are different in terms of the surface markers, cytokines and chemokines they secrete, and function (38). Similarly, HBCs cells can polarize into M1 or M2 phenotypes but they generally assume the anti-inflammatory M2 phenotype in health pregnancy (1, 39–41). Pro-M1 genes in HBCs are silenced by methylation while pro-M2 genes are hypomethylated in HBCs and thus available for transcription (2). However, some studies have shown that HBCs exhibit a diverse phenotype of both M2 and M1 (5). These cells express the arginase I and II, alongside iNOS enzymes that polarizes them towards M2 and M1 phenotypes (29, 42). Generally, M2 HBCs promote wound healing and on the contrary M1 phenotype inhibits the process (33). M2 macrophages are categorized further into M2a, M2b, M2c, and M2d (8, 21). M2a plays a role in regulating the immunity, killing of extracellular responses, and tissue repair; M2b macrophages induce Th2 responses and secrete pro and anti-inflammatory cytokines thus they can suppress and regulate inflammation. M2c macrophages are more capable of reducing inflammation, matrix deposition, and tissue remodeling, while M2d macrophages are engaged in angiogenesis and immune suppression (21, 33, 36, 43). Despite M2b having an M2 phenotype, they are similar to M1 macrophages in that they can produce pro-inflammatory cytokines such as TNF, IL-1, and IL-6 in addition to IL-10 but they lack microbicidal action (33). In normal pregnancies, HBCs primarily exhibit an M2 or immunomodulatory phenotype which helps to promote villous growth, immunological tolerance, and vascular development in a healthy pregnancy. **Table 1** below summarizes the activation signals, cell surface markers, secreted cytokines and chemokines alongside the functions of HBCs in normal pregnancy (21, 33, 36, 44–46).

**Table 1:** Classification, features and functions of Hofbauer cells in healthy pregnancy.

	M1	M2a	M2b	M2c	M2d	References
Stimulus	IFN-γ + LPS, IFN-γ + TNF, and GM-CSF	IL-4, IL-13, Fungal and parasitic infections	Immune complexes IL-1R	IL-10, TGF-β, and Glucocorticoids	IL-6, LIF, M-CSF, and Adenosine	36, 44, 45
Cell surface markers	CD80, CD86, TLR1, TLR4, and HLA DR^- High^, IL-1R	CD163, IL-1R, MHC II, CD206, CD209, IL-RN, and DC-SIGN	CD86, MHC II	CD163, TLR1, TLR8, CD206, CD 209, and CD14	CD163, CD14, and CD85	21, 33, 46
Secreted cytokine and chemokines	IL-6, IL-8, IL-10 ^Low^/IL-12^High^, IL-23, CXCL8, IL-1β, TNF-α, IFN-γ, CCL10, CCL11, CCL5, CCL8, CCL9, CCL2, CCL3, CCL4	IL-10, TGF-β, IL-10 ^Low^/IL-12^High^, and IL-1RA	IL-1, IL-6, IL-10, IL-10 ^Low^/IL-12^High^, TNF-α, and CCL1	IL-10^Low^/IL-12^High^, TGF-β, CCR2, and Pentraxin 3	VEGF, MMP-9, IDO, IL-10 ^Low^/IL-12^High^, TNF-α, TGF-β, CCL5, CXCL10, and CXCL1	21, 36, 44
Function	Kills intracellular pathogens and tumor resistance, Microbicidal activity, and induces cell mediated (TH1 type) responses	Anti-inflammatory responses, killing of extracellular responses, cell growth and tissue repair	Induces Th2 responses and pro and anti-inflammatory cytokines that can suppress and regulate Inflammation, antigen presentation	Reduces inflammation, matrix deposition, Tissue remodeling, and immune regulation	Angiogenesis and immune suppression	21, 33, 36, 46

CCR, Chemokine receptor; CXCL, Chemokine ligand; GM-CSF, Granulocyte macrophage colony stimulating factor, HLA, Human leucocyte antigen; IFN-γ, Interferon gamma; IL, Interleukin; M-CSF, Macrophage colony stimulating factor, MHC, Major histocompatibility complex, TGF, Tumor growth factor; TNF, Tumor necrosis factor; TLR, Toll like receptor; VEGF, Vascular endothelial growth factor.

## 3 Hofbauer cells and infectious disease pregnancy complications

The role of HBCs has been studied in several pregnancy complications including chorioamnionitis, HIV, ZIKA virus infections, placental malaria, spontaneous abortion among other diseases. However, knowledge of the impact of these varying complications to HBCs population and phenotype remains rudimentary (11, 28, 33, 40). Chorioamnionitis is a major cause of preterm birth, it manifests as inflammation of the placental villi. It is caused by bacterial, viral, and parasitic infections (40, 47). Several studies have shown impaired HBCs functionality in chorioamnionitis (48, 49). However, contradicting findings exist on the impact of chorioamnionitis on the number of HBCs (50, 51). This discrepancies has been linked to the low purity of HBCs as a result of contamination by maternal macrophages (52), hence more studies utilizing harmonized isolation protocols are warranted to fully elucidate this.

Viral infections such as Zika and HIV have been shown to have teratogenic effects as a result of mother to child *in-utero* transmission (32, 53). Multiplication of these viruses in HBCs contributes to vertical transmission to the fetus (32, 53, 54). However, some studies have reported conflicting findings regarding the role of HBCs in HIV infection, with some studies suggesting that HBCs inhibit HIV transmission to the fetus (55, 56). The impact of HIV and ZIKA viruses on HBCs is not fully characterized (8, 57–59).

Additionally, human cytomegalovirus (HCMV) a major cause of the congenital infections (60), is thought to infect monocytes and macrophages (61, 62). Infection of these cells at the fetal-maternal interface interferes with tolerance and can lead to vertical transmission (63). However, despite the teratogenic effect of HCMV, the role of HBCs in this infection and the consequent effect on their phenotype is unknown. These gaps in the role of HBCs in viral infections alongside the impact of the infections on HBCs phenotype necessitates additional studies. These insights from further studies will aid in the development of placental interventions for controlling adverse birth outcomes and improving the maternal-child health care.

Placental malaria (PM) is characterized by the sequestration of *Plasmodium falciparum* parasites in the intervillous spaces of the placenta (64). It exposes pregnant women and their fetuses to adverse birth outcomes such as low birth weight, preterm birth, stillbirth, severe anemia, and spontaneous abortion (65). Despite HBCs function of maintaining the maternal-fetal tolerance in pregnancy (2, 7, 66), few studies on the response of HBCs to malaria exist as reviewed earlier (28). Hence, little is known about HBCs role in PM and the impact of PM on their functional phenotype (31). In a prior study, PM was associated with an increase in CD68+ HBCs and a decrease in the anti-inflammatory CD163+ M2 population in primigravidas (31). The authors linked these findings to the inflammatory responses of the naïve fetal immunity to PM that is associated with fetal growth restriction. Additional studies will establish whether PM modifies HBCs polarization into different M2 subtypes, quantify their intracellular cytokine production and identify the relationship between intracellular cytokines and placental macrophage activation markers. Advancing knowledge on the role of HBCs in PM and the effect of malaria on their function will lead to the development of novel interventions for preventing congenital malaria and the adverse maternal-child birth outcomes.

### 4.1 Placental tissue collection for HBCs isolation

There is no consensus in time span between delivery and collection of the placental tissue for HBCs isolation. Previously, the collection of the placental tissue for HBCs isolation had been done immediately (67–70) or within 60 minutes after delivery (3, 4, 12, 18, 31, 46, 67, 68, 71, 72), though in most studies, the collection was within 20 to 30 minutes after delivery (4, 18, 31, 72).

### 4.2 Placental tissue sampling for HBCs isolation

Hofbauer cells are isolated from the fetal side of the placenta in the placental villus (chorionic villus) (7). However, there is a gap in knowledge on the site of collection. Prior studies have sampled biopsies for isolation from either the center or periphery of the placenta or between the cord insertion site and the placental periphery (27, 31, 46). Similarly, there is lack of standardized size of the placental biopsy collected for HBCs isolation. Earlier studies utilized 10 g (73), 20–40 g (70), 60–150 g (3, 72), 2 cm by 2 cm by 1 cm (27), and 2.5 cm by 2.5 cm by 2.5 cm (2.5 cm^3^) (46) of the placental villous tissue to isolate HBCs. In addition, the depth at which to sample for HBCs in the tissue for most studies was not indicated, coupled with avoidance of placental areas with infarction, hemorrhage, and calcification.

### 4.3 Isolation of Hofbauer cells

The isolation of pure HBCs remains challenging for they are located very close to the maternal intervillous monocytes (MIMs) (18), and there is no consensus in the HBCs isolation protocol (3, 4, 28, 74). Placental cells are firmly adjoined together by extracellular matrix (ECM) proteins to form tissue. Earlier studies have utilized mechanical disintegration, trypsin/collagenase enzymatic digestions, density gradient separation by ficoll and percoll, homogenization, adherence, rosetting, and immune selection using magnetic beads to isolate HBCs for phenotypic and functional studies as reviewed previously (3, 4, 7). A membrane free placental villous tissue is minced, and then subjected to enzymatic digestions using trypsin, or collagenase, and DNase or both (3, 4, 29, 74). Tang and colleagues employed four digestions. The first and the next two digestions were done in a solution containing trypsin and DNase enzymes for 15 and 30 minutes, respectively. The last digestion was carried out in a solution containing collagenase A and DNase I enzymes for 1 hour. The resulting cells were subjected to two percoll density gradient centrifugation followed by negative immune selection purification (4). HBCs with high purity (98 -99%) and yield (130 – 200 × 10^6^ per 80–100 g of tissue) were obtained by this method. However, this protocol assumed the influence of the magnetic beads and *in vitro* culture on the phenotype and the functions of isolated cells shown in a number of earlier studies (75–77). Lasch and colleagues later modified the this method to developed another protocol for HBCs isolation that eliminated the immune selection and the *in vitro* culturing steps (3). This protocol utilized similar sequential enzymatic digestions as described (4), followed by one percoll gradient separation step and lastly cell analysis was done without culturing, this maintained the placental cells in their native state. Trophoblasts contamination was largely eliminated by the pipetting technique, although traces of these cells were still detectable (3). The purity of HBCs obtained using this method was comparable to that reported by Tang et al. (4).

The previous methods are insufficient for the isolation of HBCs (27–29, 74). They employ relatively long hours of enzymatic digestion of up to 3 hours that is unnecessary (74), and may digest surface markers expressed by HBCs further complicating their characterization (7). Also, failure to use DNase in the isolation process reduces HBCs yield and viability (74). Additionally, previous studies have shown that isolated HBCs are prone to contamination by maternal macrophages because they are extracted as total CD14^+^ cells, of which approximately 30% are maternal macrophages (27–29). To circumvent these existing gaps in earlier protocols, Appios and colleagues developed standard procedures for the isolation of HBCs from first trimester and term placentas while ensuring optimal viability (74). In this protocol, the term placental villous tissue is mechanically broken down, enzymatically digested sequentially by trypsin for 15 minutes followed by Collagenase V and DNase I for 60 minutes, and one step percoll density separation. Furthermore, high purity HBCs are obtained by fluorescence-activated cell sorting (FACS) method where common HLA allotype antibodies are added to the flow cytometry panel facilitating the separation of maternal cells (HLA-DR^+^) and fetal cells (HLA-DR^-^). The term HBCs viability of 95% obtained by this protocol was comparable to 98 – 99% reported earlier by Tang et al. (4). However, this method is only feasible for first trimester placentas, as at term both maternal and Hofbauer cells express the HLA-DR^+^ surface marker (74). Despite being one of the best method available for isolation of HBCs, it has several limitations. First, it does not specify the size of the villous tissue used or the resulting HBC yield per gram and per gestational age. Secondly, in the absence of HLA allotype mismatch between the maternal and fetal cells, HBCs purity and viability is compromised further because this protocol is limited in separating the two cell populations. Lastly, the protocol cannot be applied directly without modification of enzyme concentrations, duration of enzymatic digestion, and purification strategy when isolating HBCs from placentas of different gestational ages, owing to increasing tissue complexity and changes in surface marker expression profiles as pregnancy progresses (10, 33, 74, 78).

### 4.4 Determination of HBCs purity after isolation

Cell surface markers have different efficiency for determining HBCs purity, and no single marker is adequate for assessing HBC purity. Expression of HBCs markers varies across pregnancy (10, 33), and most markers such as CD14, CD68, CD163 are common on HBCs (3, 29). Immune selection using magnetic beads, pipetting technique, and flow cytometry techniques have been used for HBCs purification after isolation (3, 4, 29, 74). Since magnetic beads activates the cells and tempers with their native state and the pipetting technique limitation (3, 75–77), a multiparmetric approach has been recommended to achieve high-purity Hofbauer cell populations (29, 74). The inclusion of HLA allotype-specific antibodies has proven useful for discriminating Hofbauer cells from maternal macrophages in first-trimester placental tissue (74). However, this method is less applicable at term, as at term both Hofbauer and maternal cells express HLA-DR (29, 79). Other markers such as FOLR2^+^ and HLA-DR^-^ have been found to distinguish HBCs from other macrophages in first trimester (29). However, this has not been applied for term placentas. These gaps necessitates the inclusion of more markers expressed solely by HBCs at term in the isolation method so as to achieve high purity (29, 79).

## 5 Future research of HBCs in infectious diseases with a focus on placental malaria

Previous studies have not fully established the role of HBCs in the pathologies of infectious diseases in pregnancy. Owing to the easy accessibility of HBCs at delivery, their role in protecting or causing congenital infections can be well elucidated. Placental malaria (PM) burden remains unacceptably high in sub-Saharan Africa with a prevalence of up to 40% at delivery (80). It exposes pregnant women and their fetuses to adverse birth outcomes such as low birth weight, preterm birth, stillbirth, severe anemia, spontaneous abortion, congenital malaria, morbidity, and mortality (65). PM causes increased fetal HBCs response and the HBCs M2 phenotype predicts the clinical birth outcome in PM in a gravidity dependent manner (31). Hofbauer cells can be utilized as predictive marker for brain macrophages (microglial) programming and, consequently, as non-invasive biomarkers for identifying infants at risk of neurodevelopmental disorder(s) (81).

In addition, future studies will elucidate micro-environmental signals that contribute to HBCs polarization in PM, thereby offering novel insights on biomarkers for placental development in the context of an inflammatory environment. The identified PM biomarkers in maternal circulation have the potential to be leveraged towards the development of effective point-of-care tools for PM diagnosis in low-resource settings. This will significantly improve maternal and child health outcomes. Also, the relationship between phenotypic and functional characterization of HBCs and birth outcomes in infants born preterm or with low birth weight from PM positive mothers could be described. Perinatal infection and preterm delivery disrupts the HBCs phenotype and function which increases the risk of women to depression and cardiovascular diseases (71). This phenomenon shows the potential of using HBCs as biomarkers for predicting the susceptibility of PM+ women to malaria and other infectious diseases later in life. Profiling HBCs dysfunction biomarkers associated with PM will be helpful in predicting maternal and infant health (40). Such studies will provide novel insights into the properties and the roles of HBC in placental homeostasis.

## 6 Conclusion

Hofbauer cells are fetal macrophages with an activated M2 functional phenotype that regulate various biological functions in the placenta such as microbicidal activity, angiogenesis for morphogenesis, and homeostasis. Studies have shown that HBCs may play roles in both normal and pathologic pregnancy, however their role remains unclear. The current knowledge on HBCs present them as the potential targets for therapeutic and preventive interventions for placental pathologies and congenital infections. In the Hofbauer cells isolation process entails, dislodging or enzymatic dissociation of HBCs from the placental tissues followed by a combination of density gradient centrifugation, filtration, and sedimentation techniques which are based on cell size and density and the magnetic separation alongside cell purification techniques that relies on the cell surface markers. Limitations by earlier methods of HBCs isolation (3, 4, 27, 28, 74), warrants the development of a simple and reproducible universal standard isolation technique with high yield, viability, and purity without compromising their phenotype and functions. This will facilitate further phenotypic and functional analysis of the understudied HBCs thus expanding knowledge on their role in infectious diseases and exploring their potential as target for control interventions of congenital infections and mother infant adverse birth outcomes.
